# Biological, Psychological, and Physical Performance Variations in Football Players during the COVID-19 Lockdown: A Prospective Cohort Study

**DOI:** 10.3390/ijerph19052739

**Published:** 2022-02-26

**Authors:** Giulia My, Santo Marsigliante, Antonino Bianco, Daniele Zangla, Carlos Marques da Silva, Antonella Muscella

**Affiliations:** 1 Department of Biological and Environmental Science and Technologies (Di.S.Te.B.A.), University of Salento, 73100 Lecce, Italy; giulia.my@unisalento.it (G.M.); santo.marsigliante@unisalento.it (S.M.); 2 Sport and Exercise Sciences Research Unit, Department of Psychology, Educational Science and Human Movement, University of Palermo, 90144 Palermo, Italy; antonino.bianco@unipa.it (A.B.); daniele.zangla@unipa.it (D.Z.); 3 Life Quality Research Center (CIEQV), Sport Sciences School of Rio Maior, Polytechnic Institute of Santarém, 2000-044 Santarem, Portugal; csilva@esdrm.ipsantarem.pt

**Keywords:** COVID-19 lockdown, hematological parameters, psychological stress, cortisol, testosterone, physical performance, football, Serie A

## Abstract

This prospective cohort study aimed to evaluate whether COVID-19 lockdown caused biological, psychological, and/or physical performance variations in footballers. We compared the 2018/2019 and 2019/2020 seasons evaluating the plasma volume, hematological parameters, iron/ferritin, creatine kinase, vitamin D, cortisol, testosterone, and physiological state of players of the Italian football major league (Serie A). Measurements were performed before the preparatory period (T0), at the beginning (T1) and in the middle (T2) of the championship, and in March (T3) and at the end of season (T4). The results showed that in the 2019/2020 season affected by the lockdown, the weight, BMI, and fat mass percentage were higher than in the previous season. Hematocrit, hemoglobin, red blood cells, and ferritin decreased during both seasons, more significantly than in the regular season. During both seasons, creatine kinase increased from T2 whilst iron concentrations decreased in T3. Testosterone increased in both seasons from T0 to T3 and returned to initial levels at T4; cortisol increased in T2 and T3 during the 2018/2019 season but not during the COVID-19 season. Physical performance tests revealed differences associated with lockdown. Thus, although from a medical point of view, none of the evaluated changes between the two seasons were clinically relevant, training at home during lockdown did not allow the players to maintain the jumping power levels typical of a competitive period.

## 1. Introduction

On 11 March 2020, the World Health Organization (WHO) officially declared a pandemic status, caused by the new coronavirus (SARS-CoV-2). The official name given by the World Health Organization to the syndrome caused by the virus is COVID-19 (short for Coronavirus Disease—2019). The pandemic of the viral disease caused by the new coronavirus is still ongoing and returning to normal activities is still a challenge [1].

The need to reduce the risk of disease transmission has also had a huge impact on sport and exercise in general. Team sports activities have been suspended, starting from the football championships up to the pinnacle of sporting excellence, the 2020 Olympic Sports Games. It emerged, in fact, that even elite athletes, who are already normally under competitive stress, are affected by physical and mental conditions consequences of the COVID-19 pandemic [2]. This pandemic-induced mental stress for elite athletes originated as early as the cancellation or postponement of matches, the ban on training, and the frequent removal and placement of blocks, also generating uncertainty for their athletic career [3,4].

After lockdown, football leagues in Europe have faced a congested schedule with multiple matches per week and short recovery periods to complete the season. This hampered the adequate preparation of players [5], leading to lower physical performance [6] and increased mental stress [7] in the season continuation after the COVID-19 lockdown. The incidence of injuries does not seem to have changed significantly with the return-to-play after the first COVID-19 lockdown in Italian professional soccer players; however, the schedule congestion and changes to the pace of the game seem to have revised the epidemiological data to date [5]. To minimize overtraining and/or injury risks in such periods, football players need the best individual assessment of their health.

Therefore, the purpose of this study was to evaluate any changes in the metabolic, muscle, and hormonal responses of elite-level football players during two consecutive seasons: the pre-pandemic season 2018/2019 and the following one (2019/2020) affected by the COVID-19 pandemic.

## 2. Materials and Methods

### 2.1. Design

**Figure 1:**
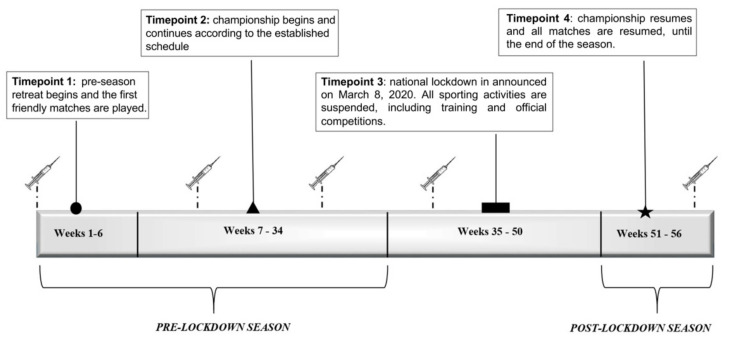
A timeline of the 2019/2020 Serie A season surrounding the impact of the COVID-19 pandemic lockdown.

This was a prospective cohort study performed in a professional elite football club in the Italian Premier League during the 2018–2019 and 2019–2020 seasons up until after the lockdown due to the COVID-19 pandemic (Figure 1). All players were evaluated five times during the study: i.e., T0, T1, T2, T3, and T4. As shown in Figure 1, T0 was scheduled before the start of the preparatory period (week 0; middle July); T1 was about at the beginning of the championship (week 14; October); T2 was programmed in the middle of the championship (week 25; January); T3 was in March (week 35); and T4 was at the end of the season (week 51; June).

### 2.2. Participants

Twenty-four players (aged 22–35 years) from an Italian Serie A football team, participating in both the 2018/2019 and 2019/2020 season, were recruited. As exclusion criteria, samples of blood were not taken from the player if they had any injury during both seasons; players that tested positive to COVID were also excluded. Goalkeepers were excluded due to their specific role in the team. Athletes who attended less than 85% of the scheduled training sessions and matches were also excluded from the study. In total, 17 players were eligible for inclusion in this study.

### 2.3. Training Program

Table 1 shows the training program followed by the team during the two seasons, differentiated according to the number of games played during the week (one or two). This program was the same in both seasons, excluding the COVID-19 lockdown, imposed in Italy from 9 March, 2020 to 3 May, 2020.

The number and duration of training sessions throughout the study were the same for all players. The duration of each training session was 90 min for all players. All training sessions were preceded by a standardized warm-up of 5–15 min.

For weeks where only one match was played on Sunday, the training protocol included sessions on Tuesdays, Wednesdays, Thursdays, Fridays, and Saturdays. Monday was instead set as a day off.

In the weeks in which two games were played (e.g., Wednesday and Sunday), the protocol included training sessions on Mondays, Tuesdays, Thursdays, and Fridays.

During training, football players reach average heart rate values (HR) of 146 beats/min corresponding to approximately 87–97% of the maximum heart rate.

Home-based training during lockdown was performed to maintain players’ physical performance levels by programs individually provided by the team’s coaches.

### 2.4. Anthropometric Evaluation

The height of the participants was measured with a Seca stadiometer to the nearest 0.1 cm, while the weight was measured with an Omron balance to the nearest 0.5 kg. Anthropometric-determined measurements included: height (m), weight (kg), body mass index (BMI (kg/m^2^) = weight/height^2^), percentage of body fat (BFP, %), and fat-free mass (FFM, kg). In particular, the percentage of body fat was estimated, following the measurement of three skin folds (chest, abdomen, and quadriceps) with a GIMA mechanical skinfold meter, using the formula developed by Jackson–Pollock [8]. The percentage of fat-free mass was measured using a bioimpedance analyzer (BIA-AKERN EFG). At each timepoint, anthropometric assessments were also carried out in the early morning, always before each workout.

### 2.5. Blood Parameters

Venous blood samples were taken following fasting in the early morning (8.00 am) following a day off. Blood (10 mL) was collected in vacutainer tubes, using an anticoagulant. The freshly drawn blood was immediately centrifuged at 3000 r·min^1^ (825 g) for 10 min to remove the plasma. Analyses were performed using a coulter blood counter (Model S-plus II, Coulter Electronics inc., Hialeah, FL, USA) and yielded values for hematocrit (Ht), hemoglobin (Hb), red blood cells (RBC), serum iron, and ferritin.

Percentage changes in plasma volume during the study period were assessed by the method described by Saidi et al. (2019) [9].

For the total 25(OH)D measurement, an Abbott Architect 25-OH D reagent on an i2000 Architect analyzer (Abbott Laboratories, Abbott Park, IL 60064, USA) was used with a chemiluminescent competitive delayed phase immunoassay (Chemiflex) standardized according to the NIST SRM 2972 (National Institute of Standard and Technology Standard Reference Material 2972). As previously reported [10], serum testosterone and cortisol were analyzed by the IMMULITE 2000 Immunoassay System (Medical Systems).

Intra- and inter-assay coefficients of variance for cortisol were 4.6% and 7.6%, respectively. The intra- and inter-assay coefficients of variance for testosterone were 3.7% and 5.6%, respectively. Serum testosterone and cortisol reference ranges were 10–75 ng/dL and 7–25 g/dL, respectively.

Normal iron storage (ferritin > 110 µg L^−1^, Hb > 14 g dL^−1^), iron depletion (ferritin < 30 µg l^−1^, Hb > 14 g dL^−1^), iron deficiency (ferritin < 12 µg L^−1^, Hb > 14 g dL^−1^), and iron deficiency anemia (ferritin < 10 µg L^−1^, Hb < 14 g dL^−1^) were defined according to population references for iron status measures in males, 24.25.

### 2.6. Physical Performances

To obtain information regarding the physiological status of youth players, we used tests that have been frequently used in similar studies: countermovement jumps test (CMJ) and Mognoni test.

To minimize any effects of diurnal variation, the three testing sessions were conducted within 2 h of the same time of the day.

Then, each player performed maximal CMJ on the contact platform from a standing position and with the hands on the hips. At the start, the subjects made a preparatory movement: from the extended leg position, they made a rapid bending of the knees until reaching the 90° angle, keeping the heels in contact with the ground and the trunk erect. After the jump, keeping the hands on the hips, the fall was performed with the knees extended, on the tip of the toes with subsequent cushioning to avoid trauma.

The ground reaction force generated during these vertical jumps was estimated with an ergo jump (Opto Jump Microgate, Bolzano, Italy). The height of the jump (cm) was the maximal height reached during the flight phase.

The Mognoni test is a simple method to evaluate the speed at which the athlete reaches OBLA (Onset of Blood Lactate Accumulation).

The test execution protocol provides that the subjects must travel 1350 m in 6 min, maintaining a constant speed of 13.5 km/h [11]. In the field version of this test used in the present study, pins were placed in the path, at regular 50 m intervals, causing players to hear a sound that informed them when the transition at each pin should take place. Immediately upon completion of the 6 min run, the capillary blood lactate concentration was measured from the earlobe with a portable lactate analyzer (Lactate Plus; Nova Biomedical, Waltham, MA, USA): the lower the value of lactate after the test, the better the aerobic fitness level [11].

### 2.7. Statistical Analysis

Results obtained were stored in Microsoft Office Excel 2016 and statistically analyzed by GraphPad PRISM 5 software (GraphPad Software). All variables used in this study were checked for the normality of distribution before the analyses (Kolmogorov–Smirnov tests). Student’s paired *t*-test and Spearman correlation were used. *p* < 0.05 was accepted as a level of statistical significance. All data obtained from the study were expressed as mean ± standard deviation.

## 3. Results

### 3.1. Anthropometric Characteristics of Football Players

The anthropometric characteristics of the players (weight, height, body mass index, percentage of body fat BFP, and fat-free mass FFM) measured during the two seasons analyzed are shown in Table 2.

The reported values show some statistical differences (*p* < 0.05) between the various points of the season. In addition, in the 2019/2020 season (COVID-19), there were higher weight values (*p* = 0.01) and BMIs (*p* = 0.03) and lower percentages of FFM (*p* = 0.01), due to the forced stop period.

**Figure 2:**
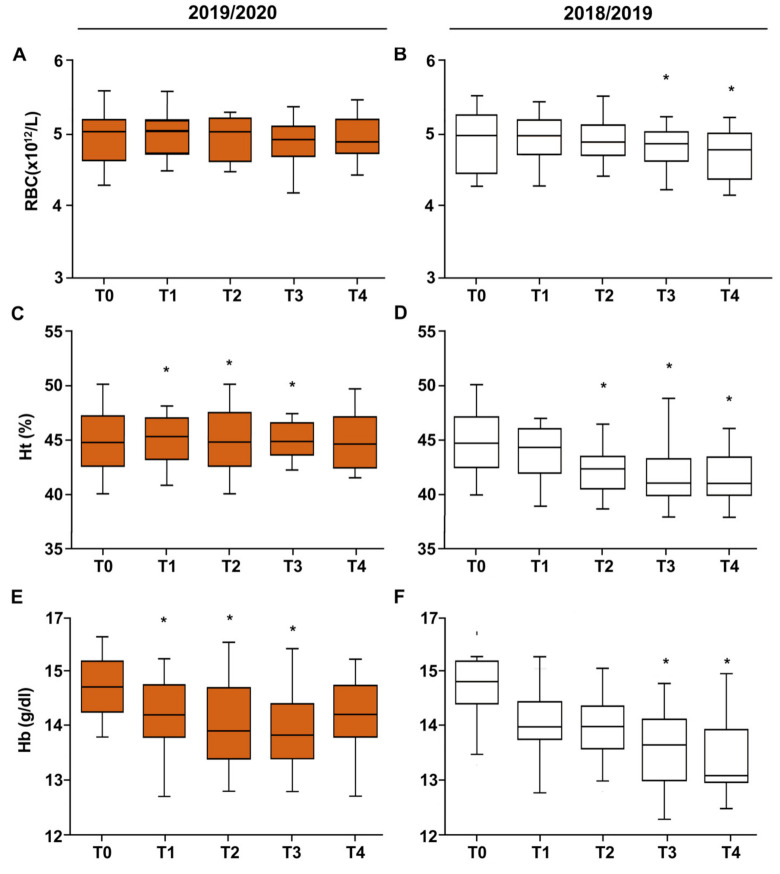
The effects of training on serum red blood cells (**A**,**B**), hematocrit (HT) percentage (**C**,**D**), and hemoglobin concentration (**E**,**F**) in football players during the 2018/2019 and 2019/2020 seasons. Box and whiskers representation of red blood cells, hemoglobin concentration, and hematocrit (HT) percentage evaluated five times (T0, T1, T2, T3, and T4) during the seasons. In this representation, the central box covers the middle 50% of the data values, between the upper and lower quartiles. The bars extend out to the extremes, while the central line is at the median. *p*-values were obtained by Student’s paired *t*-test between each timepoint and T0. * *p* < 0.05.

Exercise is known to affect hematological variables: some studies have reported a stimulation of erythrocytosis with a consequent reduction in the values of HT and HB in athletes [12]. However, it is also known that excessive physical exercise induces the physical destruction of red blood cells, also causing decreases in HT and HB [13,14]. Thus, we monitored these hematological variables, and the values measured were within the physiological ranges (4.2–5.6 × 10^12^/L for RBC; 13.0–17.5 g/dL for HB; 37–54% for HT). However, decrements in HB, RBC, and HT during both seasons (Figure 2), mostly significant in the 2018/2019 regular season were observed (Table 3).

### 3.2. Vitamin D–Iron–Ferritin

**Figure 3:**
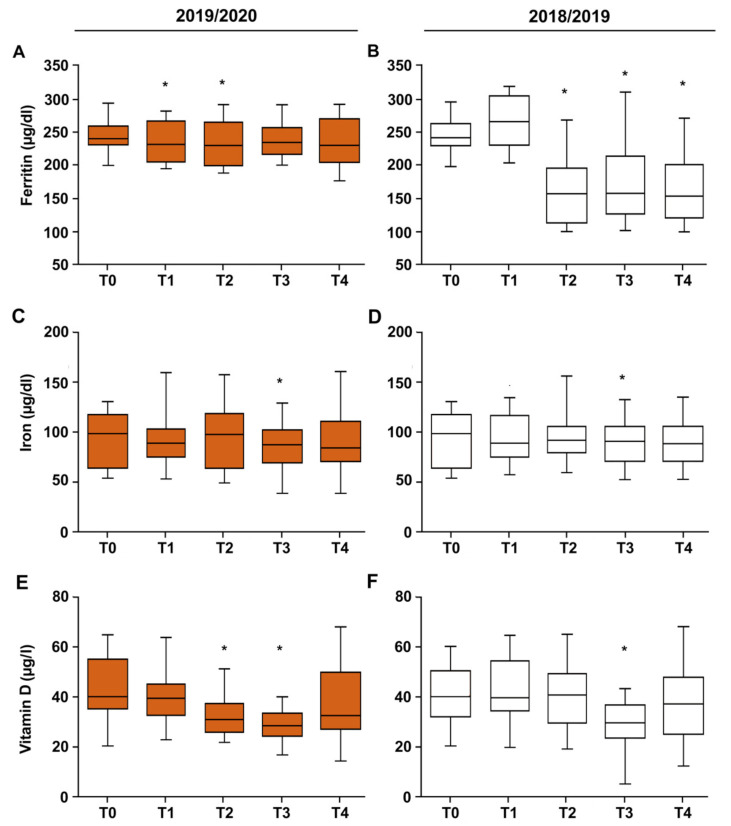
The effects of training on serum ferritin (**A**,**B**), iron (**C**,**D**), and vitamin D (**E**,**F**), concentration in football players during the 2018/2019 and 2019/2020 seasons. Box and whiskers representation of serum ferritin, iron, and vitamin D evaluated five times (T0, T1, T2, T3, and T4) during the seasons. In this representation, the central box covers the middle 50% of the data values, between the upper and lower quartiles. The bars extend out to the extremes, while the central line is at the median. *p*-values were obtained by Student’s paired *t*-test between each timepoint and T0. * *p* < 0.05.

In both seasons, vitamin D significantly decreased in T3 (<30 ng/dL; *p* < 0.05); in the 2019/2020 season (COVID-19), only vitamin D levels were also low in T2 (Figure 3 and Table 4). Cross-sectional studies indicated an association between low vitamin D concentration and low iron state [15], but we found no association (*p* > 0.05 by Spearman’s rank correlation). Ferritin levels decreased in T1 and T2 during the 2019/2020; however, a major decrement was observed in T2-T4 periods during the 2018/2019 season. Iron concentrations decreased in T3 in both seasons (Figure 3 and Table 4).

### 3.3. CK–Cortisol–Testosterone–T/C Ratio

**Figure 4:**
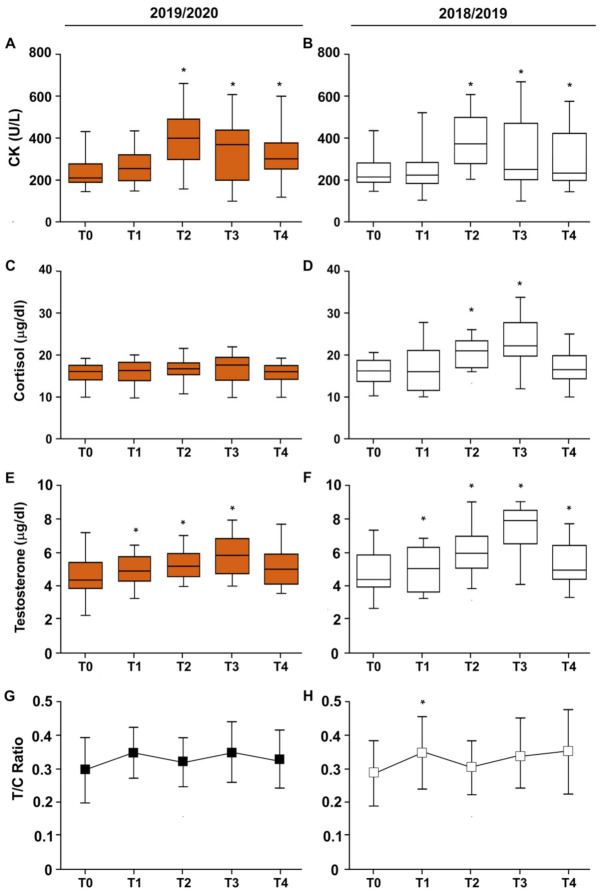
The effects of training on serum CK (**A**,**B**), cortisol (**C**,**D**), testosterone concentration (**E**,**F**), and T/C ratio (**G**,**H**), in football players during the 2018/2019 and 2019/2020 seasons. Box and whiskers representation of serum CK, cortisol, and testosterone concentration and T/C ratio evaluated five times (T0, T1, T2, T3, and T4) during the seasons. In this representation, the central box covers the middle 50% of the data values, between the upper and lower quartiles. The bars extend out to the extremes, while the central line is at the median. *p*-values were obtained by Student’s paired *t*-test between each timepoint and T0. * *p* < 0.05.

During both seasons, CK increased starting from T2 to the end of the seasons (Figure 4A,B and Table 5). As reported previously [10], cortisol concentrations increased significantly in T2 and T3 during the 2018/2019 season; nevertheless, such an increment was not found during the 2019/2020 (COVID-19) period (Figure 4C,D and Table 5).

Testosterone concentration increased in both seasons from T0 to T3 and, at the end of the season, it decreased toward initial levels; although such a decrement was statistically significant in both seasons (*p* < 0.05), it was higher in the 2018/2019 season (Figure 4E,F).

The testosterone to cortisol ratio (T/C) increased only in T1 of the 2018/2019 season; in all other periods, it did not show significant changes (Figure 4G,H).

### 3.4. Electrolytes

The clinical chemistries shown in Table 6 represent common clinical chemistries used to monitor clinical aspects of electrolytes and metabolism.

### 3.5. Physical Performances

No significant changes were observed for the CMJ Test (Table 7). However, the between-period comparison revealed significant differences because the changes associated with the COVID-19 lockdown were significantly worse than those occurring during the 2018/2019 competitive season (Table 7).

Analysis of blood lactate concentrations during the competitive seasons showed a significant decline, but a significant increase was observed following COVID-19 lockdown. Consequently, between-period differences were significant when the COVID-19 lockdown period was compared with the 2018–2019 competitive period (Table 7).

## 4. Discussion

Due to physiological and performance adaptations to training, professional football players are subjected to several alterations in health [16,17] and performance [18] throughout the course of the season. Although a variety of research has investigated performance testing and/or observational approaches to explore the relationship between training load and training outcomes (e.g., acute responses, chronic responses, and injuries) [19], limited information is available regarding blood parameters (e.g., iron storage and hormonal environment) of elite football players from the same team, before and after a different period of match play and training, even more since March 2020, when the COVID-19 pandemic forced most activities in Italy, including football, to stop. During lockdown, players could only train at home, with limited evidence regarding the effect of this period [20]. Therefore, this study aimed to investigate the effect of COVID-19 lockdown on professional football players. Thus, we describe the seasonal changes in anthropometric and body composition indicators, hormonal status, and performance in a professional football team, during two different sporting seasons: the regular 2018/2019 season and the 2019/2020 season that was stopped from March to May and finished on 2 August 2020.

Data from anthropometric, blood values, and hormonal parameters showed differences between the two seasons, proving that the forced stop period affected the physical and physiological state of professional football players. According to numerous studies, anthropometric and body composition indicators are important factors, which can predict the specific footballer’s performance, already in his adolescence [21]. In the 2018/2019 season, without any interruption, there was a constant decrease in weight, BMI, and percentage of fat mass and an increase in fat-free mass. In the 2019/2020 season that underwent the forced stop, however, the same trend was recorded but up to T1. In T2 and T3 periods, there was an increase in weight and BMI, while the percentages of fat mass and lean mass remained almost constant.

It is well established that training may alter homeostasis, hematological parameters included. Hb is a key determinate of oxygen transport and consumption [22], which is related primarily to aerobic capacity [23]. Elevated Hb is generally associated with an increase in blood oxygen transport capacity, while an increase in Ht increases blood viscosity [24]. Thus, it seems beneficial to monitor football players’ Hb and Ht parameters. Several studies showed decreases in Hb and HT values after periods of intense training or competition [25]. These declines are known as an adaptation to training [26]: erythrocytosis during exercise induces an increase in the absolute concentration of Hb [16], but this mechanism is masked by a rise in plasma volume (PV) [27]. PV expansion compensates for the negative effects of acute blood concentration induced by intense training. In fact, an increase in aldosterone levels and osmotically active plasma proteins, as well as a decrease in the activity of urodilatin, eventually lead to fluid retention and PV increment [28]. This increase in PV is a first sign of overtraining [27].

To date, only a few studies have evaluated Hb and HT values during an entire competitive season in football players [9,29]. Particularly, Silva et al. [30] observed that the Hb and HT of Brazilian football players increased significantly after 12 weeks of training. These authors postulated that such alterations were due to the plasma volume decrement observed after the football-training program. A study of Saidi et al. [9] suggested a significant change in various hematological parameters with negative effects on physical fitness during 6 weeks of congested match play. In contrast, Heisterberg et al. [31] and Rago et al. [29] recorded no significant changes in Hb and HT levels over a 6 month period in which the training and match load varied considerably. Ostojic et al. [32] found a significantly higher HT at preseason compared with other sampling periods, and no other differences were found between any of the hematologic variables during the whole season. These differences in the results obtained in these studies could be due to psychological factors, players’ diet [33], and/or differences between players’ effective match time.

In the present study, we hypothesized that in the period of congested games (between T3 and T4), in which the championship resumed after the COVID-19 lockdown, the football matches would have negatively affected the plasma volume and hematological parameters. Instead, apart from a slightly decreased Hb during the COVID-19 season, we did not find significant changes in the hematological variables tested. In addition, it should be remarked that during the COVID-19 season (i.e., when the training load is discontinuous), the hemodilution was absent. This phenomenon observed during the 2018/2019 season could be a favorable adaptation to training because decreased blood viscosity allows greater cardiac output. Instead, erythrocytes, Hb, HT, and plasma volume values decreased during the 2018/2019 season, without any interruption. Regarding erythrocytes, in agreement with our results in the 2018/2019 season, many studies have suggested that the number of erythrocytes decreased at the end of a competitive period [9]. In general, erythrocytes, Hb, and HT decrease after endurance training [34]. This is mainly caused by PV expansion [35]. In fact, Silva et al. [30] were able to show that the altered percentage of erythrocytes significantly correlated with plasma volume change (i.e., reductions) during the 12 week football training program. Among others, adequate serum iron levels seem to be the main factor for optimal hemoglobin production, maximal oxygen uptake (VO_2_max), and high sports performance in football [36]. Several investigators suggested that the iron status of elite athletes also varies during the season because of different training regimes [37]. The iron depletion in top-level football players based on low serum ferritin levels could be crucial for predicting optimal physical performance [38]. In fact, it seems that ferritin values decrease with the training load, suggesting that ferritin could be a marker of training tolerance in endurance athletes [39]. In accordance with other studies [39], in our study in footballers, ferritin showed a constantly decreasing trend from the initial phase of both seasons. Thus, the serum ferritin level strongly decreased, especially during the first part of the regular season (T1–T2). Iron is essential for normal cell biology. However, excess iron might be potentially harmful, as it can catalyze the formation of toxic reactive oxygen species. Therefore, the decreased levels of serum iron, observed along both seasons considered, might be an adaptive response [40].

Vitamin D, mainly synthesized by the skin when exposed to ultraviolet B radiation (UVB), is involved in several physiological processes as the maintenance of calcium, phosphate, and iron homeostasis [41]. During winter, vitamin D deficiency can occur in up to 50–80% of the population [42]. Although an optimal vitamin D level helps to maintain the musculoskeletal system efficiency [43], studies on athletes highlighted a surprisingly high prevalence of vitamin D insufficiency, both in outdoor and indoor disciplines [44]. Thus, even outdoor training (such as by football players) is not protective against vitamin D deficiency [45] and many studies performed in European football players during the winter season showed serum vitamin D levels below the normal range (defined by the latest guidelines as 30–50 ng/mL) [46,47]. Our data confirmed the high prevalence of low serum concentrations of vitamin D in professional male athletes [48] in winter. In both seasons, the mean concentration of Vitamin D was insufficient (defined as a serum level of 20–30 ng/mL) in T3 (in March) and in the COVID-19 season also in T2. Surprisingly, this was also true in the 2018/2019 season, when football players trained outdoors 2 h a day.

Creatine kinase (CK), cortisol, testosterone, and the testosterone/cortisol ratio (T/C) have been used to assess athletes’ response to training load [49,50]. Precisely, CK levels have been used to monitor muscle damage and post-match neuromuscular fatigue in elite football players [51] and other sports [49,50]. Most studies have reported data from single-match experiments [52] or short-term studies [53] and much less information is available on long-term studies during the entire season in elite professional football [54]. In the present study, in T2, T3, and T4 of both seasons, CK values were slightly above 270 U·L^−1^, set as the highest reference value for the general population. High values of CK have been suggested as a symptom of overreaching or overtraining [55] and unusually higher values of CK have been routinely measured in blood samples from football players (until 1492 U/L) [55]. This may be related to the nature of football training and playing involving a great deal of weight-bearing activities, which include eccentric (lengthening) contractions of the leg muscles [55]. In addition, football playing can induce muscle damage due to mechanical impact with other players. Finally, football training and competition are often performed under severe environmental conditions, and football games are among the longest (90 min) and most energy-demanding sporting activities [56].

Plasma cortisol and testosterone are sensitive to training periods that differ in volume and intensity and to the frequency of matches during the competitive period [10]. Many factors influence this delicate hormonal balance, not only training workloads, training schedules, and competition factors but also psychological stress. If the physical demands of training and competition are too great, one might assume that catabolic activities will predominate. However, when the body successfully copes with the demands, the anabolic metabolism can improve the performance during different periods of the competitive season [57]. Thus, testosterone and cortisol values can be considered important parameters that help to evaluate the influence of these factors because of the balance between anabolic and catabolic processes [58]. Some studies have identified a significant increase in cortisol concentration, in football players [10,59], due to an increased training intensity. This is probably caused by hyperresponsiveness of the hypothalamic–pituitary–adrenal (HPA) axis due to a physiological adaptation of the neuroendocrine system to training [60]. In addition, a substantial increase in cortisol, at the end of the football season, may be explained by tiredness due to the higher number of matches placing a higher physical load, as well as psychological pressure, on the players [61]. The results obtained from this study show that the hormonal concentrations of cortisol and testosterone are higher during the 2018/2019 season, in which the football players trained regularly and played all the official matches. The increase in plasma cortisol during the 2018/2019 season may represent a typical homeostatic adaption process to the soccer training and to a stressful environment and competitions [10], with an increment by 20% during the T0–T2 timepoint. In contrast, other studies have shown a significant decrease in cortisol concentration after long periods of training in elite football players [16,62]. In accordance with both Saidi et al. [9] and Requena et al. [18], we observed no changes in cortisol concentration in elite football players, during the 2019/2020 season.

Though we would have expected a significant increase in plasma cortisol after the lockdown, it should be noted that the absence of a significant change in its concentration could be attributed to the lack of stress due to the weekly official sports competitions, rather than the high level of fitness of the players and/or their usual high daily and weekly training volume over the course of the season. Several studies have documented the effects of COVID-19 on the psychological stress experienced in the general population [63], and it is known that uncontrolled stressors activate the HPA axis: through the association of the cortex, amygdala, hippocampus, and adrenal glands, blood cortisol increases [64]. As high levels of stress can have a detrimental impact on everyday life and mental and physical health, there would be a need to examine and diagnose psychological problems and deteriorating mental health among professional athletes during the COVID-19 pandemic. In fact, training restrictions and competition avoidance, due to COVID-19 lockdown, decreased competitions performance, substantiating the contribution of cognitive distress to the overall perception of effort and to performance outcome [20]. However, previous studies have reported that COVID-19 preventive measures did not provoke changes in the levels of anxiety, stress, and symptoms of depression in professional footballers [65,66]. Our study confirmed that the professional players cope very well with the changes due to COVID-19, with no noticeable changes in cortisol, indicating good psychological adaptation. Therefore, from a practical point of view, training regimens and healthy behaviors during pandemic crises could be introduced as standard habits for health and well-being. In the pandemic period in response to social distancing, coaches and athletes had to adopt a constructive problem-solving attitude and make structural changes to the training environment. Although the time spent for sport-specific training was reduced, individualized home-based training was implemented; this could have turned into improved training conditions. Indeed, at the Tokyo 2020 Olympics, swimmers’ performance trend was maintained despite the unprecedented characteristics of the previous period of preparation, demonstrating that the supposed effects of the COVID-19 lockdown on elite athletes’ performances were not apparent. Financial, social, psychological, scientifical, and technological support environments of Olympics participants could safeguard the subsistence of performances even in the case of periods of difficulty never faced before [67].

The change in anabolic hormonal concentrations could be due to interactive modifications of various endocrine parameters, and to the effects of modifications of the hypothalamic–pituitary axis on the testicles and adrenal glands. The training-induced increases in serum testosterone reflect the anabolic activity increment related to the volume of strength training and to physical performance improvements [68]. In our study, we observed that after an intense training period, testosterone concentrations increased in both seasons, remaining in normal ranges, as also reported by others [10,69]. However, decreases in testosterone have also been reported after long-term football training, perhaps due to excessive training intensity, accumulated fatigue, or physical fitness declines [9]. The testosterone concentration decrement found in T3 during the COVID-19 season might be due to tiredness caused by the high number of matches played and to the psychological pressure at the end of the lockdown period. Regarding the T/C ratio, results showed an increase from T0 to T1, during the 2018 season. According to some studies, the T/C value increment is indicative of a good training setting and can reflect physical performance improvements [70,71]. However, in contrast to these investigations, other recent studies showed a significant decrease in the T/C ratio in professional football players [9,61], as being due to neuromuscular fatigue caused by an increase in training intensity that might be related to physical performance declines [9]. The divergence of these results can be explained by differences in training programs (frequency, duration, and intensity) and/or participants’ expertise level (training history). These two hormones appear sensitive to the intensity and volume of football training and other factors such as fatigue or mood and, if properly interpreted, could provide a tool for monitoring workload and fitness. Despite the least amount of work carried out during the COVID-19 period, aerobic fitness, as measured by the Mognoni test, improved after the lockdown. There was also a significant difference between the same timepoints of COVID-19 and 2018–2019 seasons.

In addition to training volume, training intensity is another key factor that may influence aerobic adaptations [24]. Specifically, high-intensity interval training can elicit significant improvements in highly trained athletes, whereas additional submaximal endurance exercise does not seem to lead to further changes [23]. Additionally, in normal circumstances (i.e., during the competitive period), the training volume is not entirely dedicated to the improvement of players’ physical capacities, because technical-tactical drills are performed more frequently. This kind of exercise may not always reach an adequate intensity to elicit positive adaptations, whereas during the COVID-19 lockdown period, training activities were mainly focused on physical conditioning, which, in turn, probably induced superior positive aerobic adaptations.

The findings from this study are related to only one Italian football team, but different restrictions in other countries might have had different effects on football players’ physical performance and psychological implications.

In addition, different home-based training strategies (including the different equipment) could result in different adaptations, in biological and physical performance, on football players.

## 5. Conclusions

This study was conducted to provide reference data in professional football players, especially under stressful conditions, such as the pandemic period. Despite the spontaneous variability in most parameters, there were significant changes during both seasons in hematocrit, hemoglobin, iron, ferritin, vitamin D, and testosterone.

However, from a medical perspective, none of the changes were considered clinically relevant to a player’s health or training status, because all were within normal values and most likely typical modulations of homeostatic concentrations in response to a stressful environment, training, and competition. In addition, this study showed that the training volume during home confinement in the COVID-19 period was probably insufficient to allow professional football players to maintain the jumping power of competitive periods. These changes might be not relevant enough to possibly interfere with clinical decisions in football players.

## Figures and Tables

**Table 1:** Weekly training schedule, divided into 2018/2019 season, 2019/2020 season (COVID-19), and lockdown period.

PERIOD	Weeks (*n*)	Weekly Volume Mean of Training + Matches (min)	Official Matches/Week (*n*)	Training Sessions/Week (*n*)	Training Distribution (%)			Training Week Activities Description
					Aerobic	Anaerobic	Other	
**2018/2019 SEASON**	50	571 (min 526–max 616)	½	5	35	20	45	~3 high-intensity technical tacticalsessions including simulated soccer matches + 2 or 3 low-intensity technical-tactical sessions + 1 or 2 speed training sessions + 1 or 2 strength training sessions in the gym
**2019/2020 SEASON** **(From week 1 to week 34 and from week 51 to week 56)**	40	571 (min 526–max 616)	½	5	35	20	45	~3 high-intensity technical tacticalsessions including simulated soccer matches + 2 or 3 low-intensity technical-tactical sessions + 1 or 2 speed training sessions + 1 or 2 strength training sessions in the gym
**LOCKDOWN** **(From week 35 to week 50)**	16		0	5	60	30	10	4 or 5 aerobic sessions performed at home with fixed devices (treadmill or bike) or with bodyweight + 2 or 3 strength training sessions using body weight and small weights + 1 or 2 running sessions, (close to home and individually)

**Table 2:** Anthropometric characteristics of football players during different times during the 2018/2019 and 2019/2020 seasons.

Parameters	T0	T1	T2	T3	T4
**COVID season**					
WEIGHT (kg)	86.3 ± 6.0	86.9 ± 6.5	86.3 ± 6.5	87.7 ± 7.4 ^§^	86.8 ± 7.0 *^,^°
HEIGHT (m)	1.78 ± 4.9	1.78 ± 4.9	1.78 ± 4.9	1.78 ± 4.9	1.78 ± 4.9
BMI (kg/m^2^)	24.7 ± 0.6	24.6 ± 0.9	24.4 ± 0,7	24.8 ± 1.0 ^§^	24.6 ± 0.9
BFP (%)	9.4 ± 1.8	9.1 ± 2.1	8.1 ± 1.2 *^,#^	7.9 ± 1.4 *^,#^	7.9 ± 1.4 *^,#^
FFM (kg)	78.5 ± 4.6	79.5± 5.3	80.5 ± 5.9	80.3 ± 6.6 *^,A^	79.8 ± 6.21 *^,A^
**2018/2019 season**					
WEIGHT (kg)	85.6 ± 2.5	85.0 ± 3.5	85.1 ± 3.2	84.5 ± 3.0 *^,#^	84.0 ± 2.7 *^,#^
HEIGHT (m)	1.77 ± 5.6	1.77 ± 5.6	1.77 ± 5.6	1.77 ± 5.6	1.77 ± 5.6
BMI (kg/m^2^)	24.7 ± 1.0	24.5 ± 0.4	24.6 ± 0.9	24.4 ± 1.0	24.3 ± 1.1
BFP (%)	8.9 ± 0.7	8.6 ± 1.1	8.5 ± 0.9	7.9 ± 1.0 *^,#,§^	7.6 ± 1.1 *^,#^
FFM (kg)	78.2 ± 2.3	79.0 ± 3.2	80.3 ± 2.2	81.6 ± 2.1 *^,#^	82.3 ± 2.0 *^,#^

* Statistical difference from T0. ^#^ Statistical difference from T1. ^§^ Statistical difference from T2. ° Statistical difference from T3. ^A^ Statistical difference from 2018/2019 season.

**Table 3:** Differences (Δ) in red blood cells and hemoglobin concentration, hematocrit percentage, and plasma volume between each timepoint in football players for 2018/2019 and 2019/2020. *p*-values < 0.05 obtained by *t*-test show statistical differences in Δ values.

	COVID Δ	2018/2019 Δ	*p*
**Erythrocytes (M/mm^3^)**			
T0–T1	0.02 ± 0.07	−0.2 ± 0.10	0.05
T1–T2	−0.04 ± 0.35	−0.04 ± 0.43	0.48
T2–T3	−0.03 ± 0.34	−0.07 ± 0.16	0.26
T3–T4	0.05 ± 0.39	−0.12 ± 0.27	0.03
T0–T4	0.01 ± 0.37	−0.25 ± 0.45	0.02
**Hematocrit (%)**			
T0–T1	0.26 ± 2.77	−0.75 ± 3.42	0.06
T1–T2	−0.15 ± 2.79	−1.74 ± 2.43	0.03
T2–T3	0.13 ± 2.96	−0.57 ± 2.81	0.22
T3–T4	0.03 ± 2.62	−0.23 ± 0.75	0.32
T0–T4	0.28 ± 3.35	−3.28 ± 3.59	0.0001
**Hemoglobin (g/dL)**			
T0–T1	−0.54 ± 0.77	−0.57 ± 0.69	0.44
T1–T2	−0.14 ± 1.00	−0.15 ± 0.72	0.49
T2–T3	−0.12 ± 0.43	−0.34 ± 0.82	0.10
T3–T4	0.29 ± 0.83	−0.32 ± 0.81	0.009
T0–T4	−0.51 ± 0.71	−1.37 ± 0.84	0.001
**Δ PV (%)**			
T0–T1	−11.07 ± 9.28	5.41 ± 10.19	0.000004
T1–T2	1.77 ± 9.66	4.73 ± 8.79	0.05
T2–T3	0.87 ± 7.38	2.78 ± 8.10	0.15
T3–T4	−1.76 ± 9.31	2.58 ± 6.89	0.06
T0–T4	−11.22 ± 9.99	15.27 ± 10.16	0.0000002

**Table 4:** Differences (Δ) in ferritin, iron, and vitamin D concentration between each timepoint in football players for 2018/2019 and 2019/2020. *p*-values < 0.05 obtained by *t*-test show statistical differences in Δ values.

	COVID Δ	2018/2019 Δ	*p*
**Ferritin**			
T0–T1	−8.72 ± 3.66	12.17 ± 3.84	0.02
T1–T2	−3.35 ± 3.98	−98.91 ± 6.57	0.000006
T2–T3	5.97 ± 4.00	17.35 ± 6.07	0.26
T3–T4	−1.55 ± 0.47	−14.60 ± 6.41	0.23
T0–T4	−7.65 ± 4.14	−3.89 ± 5.16	0.000001
**Iron**			
T0–T1	−3.94 ± 2.98	−1.54 ± 3.6	0.31
T1–T2	4.94 ± 3.16	1.50 ± 2.98	0.31
T2–T3	−10.20 ± 3.44	−3.96 ± 2.74	0.24
T3–T4	7.65 ± 3.79	−1.00 ± 4.90	0.14
T0–T4	−1.55 ± 3.46	−5.00 ± 3.22	0.33
**Vitamin D**			
T0–T1	−4.09 ± 1.53	−0.37 ± 1.12	0.13
T1–T2	−7.14 ± 1,12	−1.92 ± 1.83	0.10
T2–T3	−3.29 ± 9.09	−10.70 ± 1.24	0.01
T3–T4	8.10 ± 1.57	5.39 ± 1.51	0.16
T0–T4	−6.43 ± 1.57	−7.60 ± 1.65	0.16

**Table 5:** Differences (Δ) in CK, cortisol, and testosterone concentration and T/C ratio between each timepoint in football players all for 2018/2019 and 2019/2020. *p*-values < 0.05 obtained by *t*-test show statistical differences in Δ values.

	COVID Δ	2018/2019 Δ	*p*
**CPK (U/L)**			
T0–T1	35.3 ± 8.6	3.9 ± 4.2	0.05
T1–T2	135.5 ± 1.6	141.8 ± 1.6	0.43
T2–T3	−64.8 ± 2.13	−64.8 ± 1.7	0.50
T3–T4	−11.3 ± 2.19	−8.2 ± 5.7	0.47
T0–T4	94.7 ± 1.43	72.7 ± 1.29	0.30
**Cortisol (µg/dL)**			
T0–T1	0.1 ± 3.48	1.1 ± 6.01	0.19
T1–T2	0.7 ± 3.46	3.4 ± 6.83	0.03
T2–T3	0.2 ± 4.26	2.9 ± 6.84	0.03
T3–T4	−0.9 ± 3.69	−6.8 ± 6.21	0.0001
T0–T4	0.1 ± 3.35	0.6 ± 4.02	0.23
**Testosterone (µg/dL)**			
T0–T1	0.9 ± 1.81	1.0 ± 1.45	0.40
T1–T2	−0.2 ± 1.26	0.6 ± 1.05	0.002
T2–T3	0.5 ± 1.69	1.4 ± 1.66	0.05
T3–T4	−0.6 ± 1.66	−2.0 ± 1.89	0.007
T0–T4	0.6 ± 1.54	1.0 ± 1.45	0.14
**T/C ratio**			
T0–T1	5.1 ± 13.67	6.0 ± 15.00	0.39
T1–T2	−2.5 ± 10.84	−4.3 ± 13.54	0.30
T2–T3	2.9 ± 13.29	2.9 ± 11.70	0.49
T3–T4	−2.2 ± 10.58	1.8 ± 15.80	0.01
T0–T4	3.3 ± 10.41	6.4 ± 13.05	0.20

**Table 6:** Differences (Δ) in electrolytes concentration between each timepoint in football players for 2018/2019 and 2019/2020 seasons. *p*-values < 0.05 obtained by *t*-test show statistical differences in Δ values.

Parameters	T0	T1	T2	T3	T4
**2018/2019 season**					
Magnesium (mg/dL)	2.25 ± 0.16	1.86 ± 0.14 ^#^	2.04 ± 0.13 ^#,^*	1.90 ± 0.13 ^#^	2.15 ± 0.12 *^,$^
Sodium (mmol/L)	142.25 ± 3.60	142.29 ± 3.59	140.17 ± 4.91	144.29 ± 3.51 ^§^	141.25 ± 3.84 ^$^
Potassium (mmol/L)	4.37 ± 0.30	4.38 ± 0.29	4.57 ± 0.28	4.44 ± 0.23	4.41 ± 0.27
**2019/2020 season**					
Magnesium (mg/dL)	2.25 ± 0.12	1.94 ± 0.12 ^#^	2.03 ± 0.13 ^#^	2.05 ± 0.25 ^#,^*^,A^	2.21 ± 0.17 *^,§^
Sodium (mmol/L)	142.25 ± 3.84	142.58 ± 1.53	140.92 ± 1.32 *	143.33 ± 1.17 ^§,A^	141.96 ± 1.52
Potassium (mmol/L)	4.37 ± 0.27	4.36 ± 0.29	4.71 ± 0.33 ^#,^*	4.60 ± 0.36	4.46 ± 0.38

^#^ Statistical difference from T0. * Statistical difference from T1. ^§^ Statistical difference from T2. ^$^ Statistical difference from T3. ^A^ Statistical difference from 2018/2019 season.

**Table 7:** Differences (Δ) in blood lactate concentrations and CMJ between each timepoint in football players all for 2018/2019 and 2019/2020. *p*-values < 0.05 obtained by *t*-test show statistical differences in Δ values.

	COVID Δ	2018/2019 Δ	*p*
**Lactate—mmol × L^−1^**			
T0–T1	−0.81 ± 0.06	−0.90 ± 0.10	0.34
T1–T2	−0.86 ± 0.15	−0.92 ± 0.34	0.08
T2–T3	−0.45 ± 0.34	−0.60 ± 0.15	0.26
T3–T4	0.90 ± 0.39	−0.50 ± 0.30	0.01
T0–T4	−0.80 ± 0.37	−1.60 ± 0.50	0.03
**CMJ—height jump (cm)**			
T0–T1	1.17 ± 0.77	1.10 ± 0.69	0.44
T1–T2	1.76 ± 1.00	1.80 ± 0.22	0.49
T2–T3	0.84 ± 0.43	0.90 ± 0.82	0.10
T3–T4	0.10 ± 0.83	1.52 ± 0.81	0.02
T0–T4	−0.51 ± 0.71	3.61 ± 0.84	0.001

## Data Availability

Data available on request due to restrictions, e.g., privacy or ethical.
